# Transgender and Non-Binary Surgery Registry: Building a Patient-Focused Registry for Genital Gender Affirming Surgery

**DOI:** 10.1089/trgh.2022.0111

**Published:** 2024-10-09

**Authors:** Geolani W. Dy, Gaines Blasdel, Daniel Dugi, Christi Butler, James M. Hotaling, Jeremy B. Myers, Isak Goodwin, Rachel Bluebond-Langner, Lee C. Zhao, Cori A. Agarwal

**Affiliations:** ^1^ Department of Urology, Oregon Health and Science University, Portland, Oregon, USA.; ^2^ Division of Plastic and Reconstructive Surgery, Department of Surgery, Oregon Health and Science University, Portland, Oregon, USA.; ^3^ Department of Urology, NYU Grossman School of Medicine, New York, New York, USA.; ^4^ Department of Urology, University of California San Francisco, San Francisco, California, USA.; ^5^ Division of Urology, Department of Surgery, University of Utah School of Medicine, Salt Lake City, Utah, USA.; ^6^ Division of Plastic and Reconstructive Surgery, Department of Surgery, University of Utah, Salt Lake City, Utah, USA.; ^7^ Department of Plastic Surgery, NYU Grossman School of Medicine, New York, New York, USA.

**Keywords:** community engagement, registry, surgery, transgender, vaginoplasty

## Abstract

**Purpose:**

High quality data regarding long-term clinical and patient-reported outcomes (PROs) of genital gender-affirming surgery (GGAS) are lacking, and transgender and non-binary (TGNB) community voices have not historically been included in research development. These factors limit the utility of current research for guiding patients, clinicians, payers, and other GGAS stakeholders in decision-making. The Transgender and Non-Binary Surgery (TRANS) Registry has been developed to meet the needs of GGAS stakeholders and address limitations of traditional GGAS research.

**Methods:**

Development of the TRANS Registry occurred over several developmental phases beginning in May 2019 to present. Stakeholder engagement was performed throughout these phases, including: determination of key clinical outcomes and PROs, creation and implementation of data collection tools within the electronic health record (EHR), and development of centralized registry infrastructure.

**Results:**

The TRANS Registry is a prospective observational registry of individuals seeking vaginoplasty and vulvoplasty. The EHR-enabled infrastructure allows patients and clinicians to contribute longitudinal outcomes data to the TRANS Registry. We describe our community engaged approach to designing the TRANS Registry, including lessons learned, challenges, and future directions.

**Conclusions:**

The TRANS Registry is the first multicenter initiative to prospectively track the health of individuals seeking vaginoplasty and vulvoplasty using EHR-enabled methods, engaging TGNB community members and clinicians as partners in the process. This process may be used as a model for registry development in other emerging fields where high-quality longitudinal outcomes data are needed.

## Introduction

Amid unprecedented demand for genital gender-affirming surgeries (GGASs),^1,2^ patients, providers, and payers are challenged by interrelated barriers: (1) the lack of high-quality prospective data on GGAS outcomes and (2) the lack of patient voices in GGAS research. Transgender and non-binary (TGNB) individuals seeking GGAS desire more meaningful transparent data on surgical outcomes to guide important decisions, such as choice of procedure, technique, and surgeon.^3^ Surgeons need data on the effectiveness of operative techniques and perioperative management strategies to improve practices. Payers, including the Centers for Medicare and Medicaid Services (CMS),^4^ continue to limit or deny GGAS coverage on the basis of scant causal evidence on effectiveness and costs.^4–6^

Scientific reports on GGAS are predominantly single-surgeon retrospective case series focused on surgeon-reported assessments of outcomes and complications.^7–9^ These studies typically lack consistency, granularity, and long-term follow-up of both clinical and patient-reported outcomes (PROs).^7^ PROs, reported directly by patients without interpretation by clinicians or caregivers, are central to GGAS; these may include experiences of care, sexual function and satisfaction, gender congruence, and urinary symptoms, among others. In a field where techniques and perioperative practices are highly variable, understanding patient perceptions and priorities regarding GGAS is essential for driving clinical change.

Longitudinal observational registries can be used to generate real-world evidence on effectiveness and quality of health care services that stakeholders need to make informed decisions.^10^ In various surgical disciplines, registries have been established to examine clinical outcomes, practice trends, and health care utilization.^11–13^ By applying principles of patient-centered outcomes research, defined as research that addresses questions and outcomes important to patients,^14^ a patient-centered registry has potential to transform GGAS care by generating practice-changing evidence that is meaningful to patients.

We have designed the Transgender and Non-Binary Surgery (TRANS) Registry as a prospective, longitudinal observational registry of individuals seeking GGAS to meet the needs of GGAS stakeholders and address limitations of traditional GGAS research, beginning with vaginoplasty and vulvoplasty. Patient- and provider-entered data spanning pre-, peri-, and post-operative periods undergo automated extraction from the electronic health record (EHR). Data from participating GGAS centers are organized in a secure, deidentified central database for future analysis. This approach to automated data extraction, described by the Registry for Stones of the Kidney and Ureter,^15^ avoids reliance on costly and labor-intensive manual data entry processes and may lower barriers to collecting consistent granular data from busy surgical practices.

By gathering PRO data directly from patients, the TRANS Registry disrupts normative power relations between GGAS clinician researchers and the patient, which have limited the utility and validity of existing studies. In a field where such power dynamics have not been addressed, where patients have had to present themselves in accordance with hetero-normative and cis-normative standards to access clinical care, research data collection at the time of clinical assessment has been noted to lead to factitious presentations.^16,17^ By prospectively tracking data on patient characteristics, surgical approaches, postoperative outcomes, follow-up management, and PROs in an unbiased series of patients, with community engagement in PRO implementation, we will gather the high-quality data needed to advance GGAS delivery. We describe our multistakeholder approach to designing the TRANS Registry, including lessons, challenges, and future directions.

## Methods

**FIG. 1.**
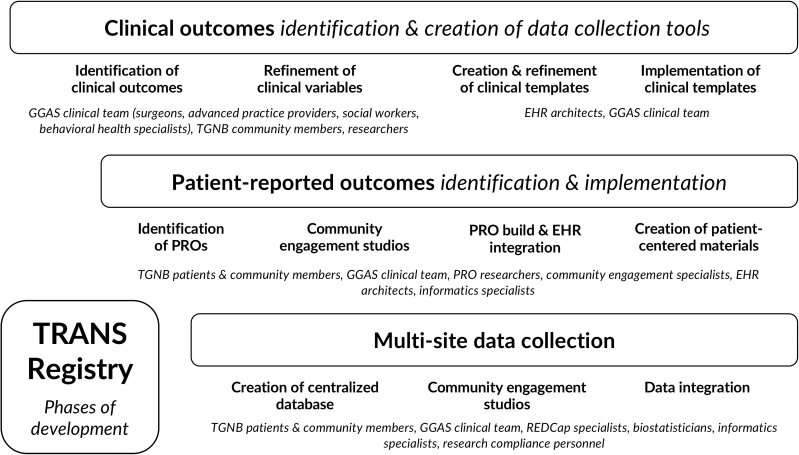
Phases and Multistakeholder Engagement in TRANS Registry Development. EHR, electronic health record; GGAS, genital gender-affirming surgery; PRO, patient-reported outcome; TGNB, transgender and non-binary; TRANS, Transgender and Non-Binary Surgery.

Beginning in May 2019, the TRANS Registry was developed over several stages (Fig. 1): determination of key outcomes, creation and implementation of data collection tools, and development of informatics infrastructure. We obtained institutional review board approval for: development of a clinical data repository (IRB no. 19743), maintenance of a multisite data repository (no. 22747), and to obtain patient consent to participate in nonclinical research questionnaires (no. 22159). Stakeholder engagement processes included: (1) clinical outcome identification through discussion groups with surgeons, researchers, and TGNB leaders, followed by variable prioritization with clinical leaders at pilot registry sites; and (2) PRO measure identification and implementation planning, through engagement of patients, PRO experts, social workers, and clinician-researchers from pilot registry sites.

### Clinical outcomes: identification and creation of data collection tools

Clinical outcome identification began with literature review and engagement of individuals with clinical experience in GGAS. Fifty GGAS stakeholders gathered at the American Urological Association—Society of Genitourinary Reconstructive Surgeons Research Session 2019 Annual Meeting in Chicago, IL for a 3-h session. Attendees included urologists, plastic surgeons, and other health care team members, as well as TGNB leaders with experience in GGAS care or provider education. The session objective was to identify relevant clinical outcomes in GGAS research. Experts described current literature and knowledge gaps regarding common GGAS procedures: vaginoplasty (e.g., surgical reconstruction of vulvar anatomy, including clitoris, labia minora, labia majora; and internal vaginal canal from penoscrotal anatomy), vulvoplasty (e.g., creation of vulvar anatomy), metoidioplasty (e.g., creation of a phallus from androgenized clitoral tissue), and phalloplasty (e.g., creation of a phallus from local or distant flaps). Attendees were then organized into facilitated discussion groups of 7–10 participants, followed by feedback sessions to gather input on clinical data and PROs in GGAS. Abbreviated agenda details and discussion questions are listed in Appendix A1.

At this time, the registry focuses on vaginoplasty and vulvoplasty, with future expansion to include penile reconstruction surgery data. To implement data collection in clinical care, important outcomes were translated into discrete variables. These variables were reviewed and defined asynchronously and over a series of virtual meetings, by a multidisciplinary group of stakeholders from pilot registry sites. Pilot sites included three academic GGAS centers chosen for their surgical volume, track records of research and clinical collaboration, multidisciplinary infrastructure, established systems for TGNB community engagement in clinical pathway and research development, and mechanisms for long-term follow-up of patients.

Given the lack of established definitions and grading scales in vaginoplasty and vulvoplasty outcomes (i.e., major vs. minor wound separation), clinicians from the registry sites participated in a consensus process for establishing definitions within the registry. These definitions are available in a manual of operations that future registry sites may reference. For areas where consensus could not be established (i.e., a subset of preoperative anthropometric measurements), individual sites will collect data independently. A patient intake form was developed to standardize collection of TRANS Registry data variables at the initial surgical consultation. This form includes data on social determinants of health; medical and transition history; and a scale of patient-reported goals and priorities of GGAS. Data instrument testing and revision took place over 12 months.

### PROs: identification and implementation

Phase 2 involved identification of scales and creation of a patient-centered plan for PRO implementation in a clinical setting between May 2020 and May 2021. Following a literature review of PROs in GGAS for core PRO selection,^9^ we consulted with surgical PRO experts, including the researchers developing GENDER-Q,^18^ and consultation with the researchers who developed PROMIS Sexual Function 2.0,^19^ who advised on adaptation of items for our population.

Two community engagement studios^20^ were held virtually in February 2021, each including 4–5 patients with vaginoplasty or vulvoplasty experience from across the United States. Engagement studios were cofacilitated by an engagement specialist, the TRANS Registry PI, and a research associate from the TGNB community. Before the engagement studios, participants were introduced to the study and provided with registry templates and PROs for review. During the 2-h virtual discussion groups, the research team described the goals and preliminary design of the TRANS Registry, including PRO workflows and data management plans. We recognized the trauma TGNB individuals endure in accessing health care, power dynamics between patients and GGAS providers, and inherent sensitivity of information on GGAS outcomes. Community partners were invited to provide direct feedback on the relevance of selected PROs, experiences of completing the questionnaires, and processes needed for the registry to meet their needs.

Following the engagement studios, participants were compensated with $100 gift cards and asked to complete a survey for feedback regarding their experiences. Transcripts were then coded by the engagement personnel, and summaries of the results were presented to the research team. We then worked with a patient consultant, team psychologist, and social worker to incorporate recommendations into registry design, PRO prompts, and all patient-facing forms and messaging to maximize transparency and patient centeredness.

### Multisite data collection and integration

PRO implementation processes were developed with input from clinicians, patients, clinical staff (i.e., patient care navigators, clinic schedulers), and informatics specialists. Clinical data collected through EPIC SmartForms and PRO questionnaires undergo data transformation to a REDCap database, to populate the registry. Our REDCap and Research Data Warehouse teams have built a mechanism for data transfer from EPIC to the HIPAA-compliant REDCap database, using this linkage to export template forms into the secure registry.

REDCap is an open source, secure, web-based application designed to support data capture for research studies, providing (1) an interface for validated data entry; (2) audit trails for tracking data manipulation and export procedures; (3) automated export procedures for data downloads to common statistical packages; and (4) procedures for importing data from external sources.^21^ REDCap is available at most U.S. university-based centers which allows for creation of a shared data dictionary for dissemination to other sites. Each site hosts an identical local REDCap database. The data are securely transferred quarterly to the centralized TRANS Registry, where the data undergo cleaning, verification, and validation.

## Results

Through stakeholder engagement, we have developed standardized TRANS Registry clinical documentation templates and a patient-centered PRO series available to all GGAS centers. Table 1 demonstrates clinical variables and PROs collected at various timepoints. Specific clinical data variables are included in Appendix A2.

**Table 1.** Variables Included in Transgender and Non-Binary Surgery Registry

	Preoperative		Operative	Postoperative	
	Initial consultation	Preoperative (after surgical candidacy established)	Operative	Hospitalization	Outpatient follow-up (1, 3, 6, 12 months postoperative, yearly)
Clinical data	Demographics Past medical history Past surgical history Social history Level of education Disability status Body mass index		Operation performed Grafts and flaps used Anthropomorphic measurements Postoperative vaginal dimensions^a^	Length of stay Inpatient complications Duration with vaginal packing^a^ Duration of urinary catheterization	Dilation practices Vaginal dimensions^a^ Postoperative events and complications
Patient-reported outcomes	Urinary distress Obstructive urinary symptoms Surgical goals and priorities	Health-related quality of life Gender congruence Sexual health Genital self-image Depressive symptoms Social support			Health-related quality of life Gender congruence Sexual health and interfering factors Genital self-image Depressive symptoms Social support Urinary distress Obstructive urinary symptoms (PROs limited to 6 and 12 months, yearly)

^a^ Not applicable for vulvoplasty patients.

PROs, patient-reported outcomes.

During the selection of PRO measures, we selected generic and condition-specific scales that were: validated in TGNB communities when possible (Transgender Congruence Scale)^22^; involved sexual and gender minorities in measure validation (Patient-Reported Outcomes Measurement Information System^®^ [PROMIS] Sexual Function and Satisfaction Measures 2.0)^19^; or were widely utilized within GGAS studies (Female Genital Self-Image Scale). For measures of mental health, quality of life, and social support, we selected PROMIS measures to allow comparison with other patient populations.

Patient partners endorsed the value of the PROs and expressed key concerns that informed our registry design: (1) certain PROs should not be accessible to the health care team and, instead, be stored directly in the deidentified registry (e.g., sexual preferences); (2) health care providers serve as “gatekeepers” to GGAS access, which informs timing of PRO administration; (3) PROs other than urinary PROs should be given after consultation and discussion of surgical prerequisites, otherwise risking inaccurate responses; (4) content warnings should accompany PROs, as even those used in standard clinical practice could trigger prior traumatic experiences and dysphoria; and (5) mental health resources should be available with each questionnaire.

Community input resulted in additional data variables (i.e., whether an individual identifies as someone with a disability), tailoring and removal of gendered language on forms, and alteration of the PRO timeline, including administration of most baseline PROs after surgical candidacy has been agreed upon by the patient and surgeon. A community consultant, team psychologist, and social worker helped to develop patient-centered explanations of each measure, guidance for accessing mental health resources, and information regarding the purpose and planned use of the data collected. Separate PROs for research purposes will be implemented through REDCap, collected outside of clinical care.

During PRO testing, we identified barriers to remote electronic implementation of mental health PROs. Our EHR systems currently lack a mechanism for alerting clinicians when a patient endorses high risk of suicidality. Because of our inability to identify and act upon concerning responses immediately, we exchanged the Patient Health Questionnaire-9 (PHQ-9), which reflects symptoms of major depression, including suicidality, for the Patient Health Questionnaire-2, which reflects depressed mood and anhedonia, to screen for depression.^23^ These clinical PROs have been integrated into standard clinical practice through the EHR (Table 2).^19,22–28^

**Table 2.** Patient-Reported Outcome Measures in the Transgender and Non-Binary Surgery Registry

PRO measures in the TRANS Registry			
Survey construct	Measure name	No. of items	Timepoints
Health Related Quality of Life	PROMIS-29^24^	12 (subset of PROMIS-29 to assess physical, mental, and social health)	Preop, 6, 12 months post-op, yearly
Sexual Health	PROMIS Sexual Function and Satisfaction Measure (SexFS 2.0)^19^	2–12	Preop, 6, 12 months post-op, yearly
Sexual Health—Interfering Factors	PROMIS Sexual Function and Satisfaction Measure—Interfering Factors (SexFS 2.0)	3	1 year post-op, yearly
Genital Self-Image	FGSIS^25^^a^	4 (most widely used genital self-image scale among transgender women)	Preop, 6, 12 months post-op, yearly
Gender Congruence	TCS^22^	12 (validated among TGNB patients)	Preop, 12 months post-op, yearly
Depressive Symptoms	PHQ-2^23^	2	Preop, 6, 12 months post-op, yearly
Social Support	PROMIS Instrumental and Emotional Support^26^	8+4	Preop, 12 months post-op, yearly
Obstructive Urinary Symptoms	IPSS^27^	8	Consult, 6, 12 months post-op, yearly
Urinary Incontinence and Distress	UDI-6^28^	6	Consult, 12 months post-op, yearly

^a^ Name adapted to “Genital Self Image Scale” for TRANS Registry based on community input.

FGSIS, Female Genital Self Image Scale; IPSS, International Prostate Symptom Score; PHQ-2, Patient Health Questionnaire-2; PROMIS-29, Patient-Reported Outcomes Measurement Information System^®^-29; TCS, Transgender Congruence Scale; TGNB, transgender and non-binary; TRANS, Transgender and Non-Binary Surgery; UDI-6, Urogenital Distress Inventory.

Requests for deidentified data are currently limited to investigators from TRANS Registry sites; however, expansion will include a mechanism for external data requests. A governance committee composed of patients, clinicians, and other GGAS stakeholders will approve requests for deidentified data. As in all clinical research, protection of patient privacy is paramount. Describing data collection, deidentification, transfer, and analysis processes was an important part of community engagement and are now explained in patient materials accompanying PROs.

Engagement of patients, caregivers, health care team members, and other stakeholders will be central to subsequent research activities, including data analysis, interpretation, article preparation, and dissemination.

## Discussion

The TRANS Registry is a unique multicenter initiative to collect consistent clinical and PRO data using patient-centered principles, to develop studies that drive clinical change. Amid rapid evolution in GGAS delivery, such infrastructure is critical for understanding patient experiences, improving peri-operative care and surgical techniques, and enhancing the quality of health care for the broader TGNB community.

Findings from our engagement process add to current literature describing methodological limitations of GGAS outcomes studies. Most studies on GGAS outcomes lack PROs, use heterogenous measures, or describe postoperative PROs without baseline data for comparison.^8,9,29^ Nearly all lack community engagement; in a systematic review on TGNB community involvement in studies on PROs of GGAS, only 5% of 158 studies included TGNB individuals as research subjects in PRO validation (using processes that did not necessarily address content validity), and fewer involved TGNB individuals in study development.^30^ As a result, patient perspectives on GGAS outcomes remain poorly characterized.

PRO data quality is influenced by factors, including measure development, administration and data collection methods, and patient-level response behaviors.^31^ Therefore, successful PRO implementation in TGNB research demands thoughtful consideration of community needs in addition to clinical, operational, and analytic expertise.^32^ Our interdisciplinary effort included surgical, medical, and behavioral health providers, informatics specialists, and researchers, while prioritizing TGNB community experiences. Our engagement demonstrated that without carefully addressing gatekeeping concerns or providing adequate context and resources, GGAS studies involving PROs may capture experiences of GGAS patients inaccurately, and they may exacerbate trauma and distress.

### Challenges and limitations

We encountered several challenges in registry development and engagement. Implementation of a clinical registry requires considerable resources and logistical coordination between clinicians, health care team members, informatics specialists, and other stakeholders, as well as extensive coordination between institutions, to ensure that all the necessary infrastructure is established before expansion.

Certain demographic elements are collected through separate patient registration processes and may be incomplete (e.g., race and ethnicity) or inaccurately reflect a patient's current status (i.e., gender identity). However due to institutional rules, we are unable to collect these data separately in registry-linked fields during clinical encounters and, therefore, must rely upon manual extraction of missing and updated data.

We endeavored to balance use of structured and free text fields to maximize granularity and consistency of data; however, with expansion to other sites, our definitions and assumptions may not be shared by other clinicians. For example, the term “vaginal prolapse” is often used interchangeably in GGAS for pelvic organ prolapse and neovaginal skin protrusion, entities with distinct etiologies, and treatments. Our clinical templates distinguish between the two and would require agreement from future users. With advancement in our collective understanding of GGAS outcomes, additional fields may be needed and definitions of various outcomes and complications revised to address this changing landscape.

A key goal of the TRANS Registry is to meet the needs of many stakeholders, while elevating patient voices and needs using a trauma-informed approach. Yet despite these efforts, our work will still be subject to pitfalls of traditional GGAS research and care provision, which exist within long-standing power dynamics and community distrust of clinician researchers.

During community engagement, white trans women and non-binary individuals were over-represented despite our attempts to recruit TGNB people of color. As engagement processes continue, we will improve representation of diverse perspectives using targeted outreach and accommodations. Utilizing such strategies in a subsequent GGAS research prioritization process successfully engaged TGNB communities who experience additional barriers to surgery (i.e., Black, LatinX, Indigenous individuals, those with lived experiences of incarceration, sex work, disabilities, as immigrants and/or refugees).^33^

Integration of PROs into the TRANS Registry is a particular challenge. At the time of PRO selection, there was no comprehensive validated PRO measure for TGNB individuals seeking gender-affirming care; however, as GENDER-Q and other validated measures^34^ become available, TRANS Registry measures may be updated. Although we anticipate that some patients may experience questionnaire fatigue, the PROs included in the registry may still be inadequate to encompass the broad range of GGAS outcomes. For example, one stakeholder expressed concern that the registry project attempts to do “too much,” and another expressed that concepts important to their GGAS experience were missing (e.g questions about eating disorders and nutrition). Overall, community stakeholders expressed that the PROs captured important aspects of their surgical experiences and endorsed the frequency of postoperative PRO administration.

These limitations notwithstanding, the TRANS Registry is the first multicenter initiative to prospectively track the health of individuals seeking vaginoplasty and vulvoplasty using EHR-enabled methods, engaging TGNB community members and clinicians as partners in the process. This process may be used as a model for registry development in other areas of GGAS where high-quality longitudinal outcomes data are needed.

### Future directions

Through the TRANS Registry, we will be able to: describe longitudinal clinical and PROs of vaginoplasty and vulvoplasty in a diverse cohort of patients, so that patients and health care providers can tailor counseling and anticipate health care maintenance needs; study which preoperative characteristics are associated with higher complication rates, so that GGAS teams may adequately prepare and support high-risk patients; understand which peri-operative management strategies are associated with postoperative complications (e.g., venous thromboembolism rates in the setting of various peri-operative estrogen management strategies, mechanical and/or chemoprophylaxis, bedrest regimens) so we can standardize highly variable practices; and evaluate how different preoperative requirements influence long-term outcomes (e.g., how different preoperative scrotal hair removal techniques impact patient experiences and long-term vaginal health), so insurers may provide better coverage for such medically necessary costly services.

Many of these basic questions are yet to be answered through high quality studies, yet may profoundly impact delivery and coverage of GGAS and ancillary services.

Although the current iteration of the TRANS Registry focuses on vaginoplasty and vulvoplasty, this infrastructure may be expanded to include individuals seeking gender-affirming penile construction. Doing so would require similar processes of stakeholder engagement to identify and define a core set of clinical variables and PROs for this patient population.

Currently, patients will have access to personal clinical outcomes data and PROs only. In the longer term, we will identify best practices for sharing findings from the larger deidentified cohort through patient dashboards and portals.

Finally, during this initial phase of TRANS Registry development, we have limited participating sites to three pilot GGAS centers. Expanding the TRANS Registry to other centers will provide important insights on patient experiences and outcomes from a broader range of surgical techniques (e.g., bowel vaginoplasty), as well as through different care models, including private practice centers and managed care organizations. Many larger scale surgical registries are managed by a governing professional society. TRANS Registry expansion will require additional infrastructure to accommodate centers with different EHR platforms, modifications to registry governance plans, and ongoing community engagement.

## Conclusions

The TRANS Registry infrastructure has been created through collaboration of researchers from the TGNB community, PRO experts, social workers, and surgeons from high-volume GGAS centers to standardize collection of prospective longitudinal surgical outcomes data. By elevating patient experiences of vaginoplasty and vulvoplasty, the TRANS Registry will provide critical insights needed by patients, providers, health care systems, and payers to guide patient-centered decision-making.

## Abbreviations Used

AUA-SS: American Urological Association-Symptom Score
Circ: circumference
CMS: Centers for Medicare and Medicaid Services
Diam: diameter
EHR: electronic health record
FGSIS: Female Genital Self Image Scale
GGAS: genital gender-affirming surgery
IPSS: International Prostate Symptom Score
N/A: not applicable
PHQ-2: Patient Health Questionnaire-2
PHQ-9: Patient Health Questionnaire-9
Post-op: post-operative
PRO: patient reported outcome
PROMIS-29: Patient-Reported Outcomes Measurement Information System^®^-29
TCS: Transgender Congruence Scale
TGNB: transgender and non-binary
TRANS: Transgender and Non-Binary Surgery
UDI-6: Urogenital Distress Inventory
VTE: venous thromboembolism

## Appendix

### Appendix A1. Agenda and Discussion Questions at AUA-GURS Meeting May 2, 2019

9:00–9:05 a.m. Welcome

9:05–9:30 a.m. Research: Outcomes and Complications

9:30–10:30 a.m. Breakout Session 1: Outcomes Reporting

- What are 3–5 outcomes that should be discussed in every article on (surgical procedure)?
- What are 3–5 complications that should be reported in (surgical procedure)?
- What are 3–5 of your biggest knowledge gaps in operative technique and/or perioperative care for patients seeking (surgical procedure)?

10:30–11:00 a.m. Patient-Reported Outcomes (PROs)

11:00–12:00 p.m. Breakout Session 2: Knowledge Gaps and Recommendations

- What information do we need from PROs to better counsel patients? How can this information be utilized?
- What domains do we need to consider?
- Who are stakeholders in PRO data?

### Appendix A2. Data Variables Collected in Clinical Templates

Data variables collected directly from the electronic health record include: past medical history, past surgical history, family history, social history, allergies, and current medications. In addition, the following variables are collected at clinical encounters.

**Table:**

Data variables		
Note	Data item	Selections
Preoperative clinic note	Social transition year	Year
	Hormone transition year	Year, N/A (no hormones)
	History of pubertal suppression	No Yes, (year)
	Prior vaginoplasty or vulvoplasty?	Yes, no
	If yes, which surgeon?	^***^
	If yes, year	
	Currently partnered	Yes, no
	Identify as having a disability?	Yes, no
	Support system	partner, family, friends, support groups, online communities, co-workers, ^***^
	In the past 30 days, how often have you been able to have an orgasm/climax when you wanted to?	0—Have not tried to have an orgasm/climax in the past 30 days 1—Never 2—Rarely 3—Sometimes 4—Often 5—Always
	Using penis for penetrative intercourse?	Yes, no, N/A, prefer to answer
	Sexual trauma history?	Yes, no, prefer not to answer
	Voiding problems	Urinary retention Urinary leakage Postvoid dribbling Other ^***^ No
	Baseline constipation?	Yes, no
	Chronic pelvic or genital pain?	No Pelvic Genital
	Uroflow:	Yes, no
	Qmax (mL/sec) max flow rate	^***^
	Qmean (mL/sec) mean flow rate	^***^
	Voided time (sec)	^***^
	Voided volume (mL)	^***^
	PVR (mL) post void residual	^***^
	Impression	^***^
	Goals of operation: have vulva	0—N/A 1—Not at all important 2—Slightly Important 3—Important 4—Fairly important 5—Very important
	Goals of operation: remove penis, scrotum	0—N/A 1—Not at all important 2—Slightly Important 3—Important 4—Fairly important 5—Very important
	Goals of operation: remove testicles	0—N/A 1—Not at all important 2—Slightly Important 3—Important 4—Fairly important 5—Very important
	Goals of operation: have receptive intercourse	0—N/A 1—Not at all important 2—Slightly Important 3—Important 4—Fairly important 5—Very important
	Goals of operation: maintain erogenous sensation	0—N/A 1—Not at all important 2—Slightly Important 3—Important 4—Fairly important 5—Very important
	Goals of operation: comfort in public settings (locker room, bathing suit)	0—N/A 1—Not at all important 2—Slightly Important 3—Important 4—Fairly important 5—Very important
	Goals of operation: relieve gender dysphoria	0—N/A 1—Not at all important 2—Slightly Important 3—Important 4—Fairly important 5—Very important
	Goals of operation: have self-lubricating vagina	0—N/A 1—Not at all important 2—Slightly Important 3—Important 4—Fairly important 5—Very important
	Other goals of operation?	None ^***^
	Housing status at the time of consult	Permanent housing Temporary housing Not currently housed
	Employment status at the time of consult	Employed Retired Student Legally disabled Unemployed
Operative report	Surgery type	Primary vaginoplasty—open perineal penile inversion Primary vaginoplasty—robotic peritoneal flap with penile inversion Vulvoplasty Revision vaginoplasty—robotic peritoneal flap Revision vaginoplasty—perineal Bowel Other ^***^
	Date of surgery	Months, date, years
	If revision, vaginal width	Unable to accommodate any dilator P1—Purple (2.2 diameter, diam/7.0 cm circumference, circ) P2—Orange (2.5 diam/8.0 cm circ) 1—Purple (2.9 diam/9.0 cm circ) 2—Blue (3.2 diam/10.0 cm circ) 3—Green (3.5 diam/11.0 cm circ) 4—Orange (3.8 diam/12.0 cm circ)
	Vaginal width- no dilator	^***^
	If revision, vaginal depth	<1 (9.7 cm)^***^ 1 (9.7 cm) 2 (10.9 cm) 3 (12.1 cm) 4 (13.3 cm) 5 (14.5 cm) >5 (14.5 cm)
	Circumcised?	Yea, no, N/A
	Stretched penile length	Stretched penile length ^***^ cm, N/A
	If scrotum, circum size (cm)	^***^ NA 12 cm (Orange #4 dilator) 11 cm (Green #3 dilator)
	If scrotum, length size (cm)	NA ^***^
	If abdomen/groin, size (cm2)	None Abdominal skin graft used: ^***^ cm2 Other skin graft: harvest site ^***^, ^***^ cm2 Peritoneum
	Final operative vaginal width	P1—Purple (2.2 diam/7.0 cm circ) P2—Orange (2.5 diam/8.0 cm circ) 1—Purple (2.9 diam/9.0 cm circ) 2—Blue (3.2 diam/10.0 cm circ) 3—Green (3.5 diam/11.0 cm circ) 4—Orange (3.8 diam/12.0 cm circ) Unable to accommodate any dilator {386273}
	Final operative vaginal depth	<1 (9.7 cm) ^***^ 1 (9.7 cm) 2 (10.9 cm) 3 (12.1 cm) 4 (13.3 cm) 5 (14.5 cm) >5 (14.5 cm) Other ^***^
	Estimated blood loss (cc)	^***^
	Intraoperative complications	None Vascular injury Bladder injury Urethral injury Rectal injury Other ^***^
	Hair removal technique	Laser (preoperative), electrolysis (preoperative), follicular destruction (intraoperative), not performed
	If vascular	^***^
	If bladder	^***^
	If urethral	^***^
	If rectal	^***^
Hospitalization data	Post-op day of vaginal packing/stent removal	N/A, 1–10, >10
	Post-op day of foley removal	1–10 and ^***^
	Post-op events and complications	VTE Postoperative bleeding requiring additional surgery Postoperative urinary retention requiring Foley replacement Other ^***^ None
Postoperative clinic note	Patient reported vaginal width/circumference (dilator color)	Not dilating, using other tools/intercourse Dilating with other device: ^***^ P1—Purple (2.2 diam/7.0 cm circ) P2—Orange (2.5 diam/8.0 cm circ) 1—Purple (2.9 diam/9.0 cm circ) 2—Blue (3.2 diam/10.0 cm circ) 3—Green (3.5 diam/11.0 cm circ) 4—Orange (3.8 diam/12.0 cm circ)
	Patient reported vaginal depth (no. of dots on dilator)	1 (9.7 cm) 2 (10.9 cm) 3 (12.1 cm) 4 (13.3 cm) 5 (14.5 cm) >5 (14.5 cm) Other estimated depth: ^***^
	Provider canal examination	Not assessed, speculum examination only
	Speculum examination	Fully inserted, partially inserted, not performed
	In the past 30 days, how often have you been able to have an orgasm/climax when you wanted to?	0—Have not tried to have an orgasm/climax in the past 30 days 1—Never 2—Rarely 3—Sometimes 4—Often 5—Always
	Able to have receptive vaginal intercourse	Yes, no, not attempted
	Comfort with dilation	1—Very comfortable 2—Comfortable 3—Neither comfortable nor uncomfortable 4—Uncomfortable 5—Very uncomfortable/unable to dilate Not dilating
	Frequency of dilation	3 times/day or more 2 times/day Once daily 4–6 times/week 1–3 times/week Every other week Once per month Less than once per month Not dilating
	Using pelvic floor PT	Yes, no, not assessed
	Post op complications—wound healing	None Superficial necrosis requiring external dressing changes Labial wound separation/dehiscence Granulation tissue Loss of flap or graft ^***^
	If necrosis	Size: ^***^×^***^ cm
	If labial wound	Size: ^***^×^***^ cm
	If granulation tissue	^***^
	If loss of flap or graft:	Partial, full
	Post op complications—canal related	None Chronic vaginal discharge >6 months post-op Inadequate canal depth/width (per patient) Stenosis at pelvic floor Eversion/protrusion of tissue at introitus Other ^***^
	Post op complications—cosmetic concerns	None Insufficient clitoral hood Buried clitoris Lack of definition of labia minora Asymmetric labia minora Flattened labia majora Asymmetric labia majora Excess skin at labia majora Other ^***^
	Post op complications—voiding problems	None Forward urinary stream/spraying Postvoid dribbling Urinary retention Urinary incontinence Other ^***^
	If incontinence:	Mixed Stress Urge
	Post op complications—persistent pain	None Pain with dilation Pain with intercourse Clitoral pain Other ^***^
	Post op complications—infection	None UTI Cellulitis Abscess Other ^***^
	If abscess, then location	^***^
	Post op complications—bleeding	None Hematoma Bleeding requiring transfusion
	Post op complications—fistula	None Vesicovaginal fistula Rectovaginal fistula Urethrovaginal fistula
	Post op complications—clitoral	None Loss of sensation Necrosis
	Post op complications—other	None Positioning-related neuropathy Ileus Small bowel obstruction Peritoneal flap dehiscence Other: ^***^
	Additional surgeries required	None Hemostasis/management of postoperative bleeding Abscess drainage Curettage/fulguration of granulation tissue in OR Meatoplasty/takedown of urethral Web Esthetic Revision of vaginal canal Repair of vesicovaginal/urethrovaginal fistula Repair of rectovaginal fistula Other: ^***^
	If hemostasis	^***^
	If abscess	^***^
	If granulation tissue	^***^
	If esthetic, meatoplasty	^***^
	If esthetic, type	Clitoroplasty revision Labia minora/clitoral hood revision Labia majora revision
	If esthetic, details	^***^
	If revision, type	Perineal approach Robotic approach Bowel vaginoplasty
	If revision, details	^***^
	If fistula	^***^

^***^ Free text options.

AUA-SS, American Urological Association-Symptom Score; Circ, circumference; Diam, diameter; N/A, not applicable; Post-op, postoperative; VTE, venous thromboembolism.

## References

[1] Canner JK, Harfouch O, Kodadek LM, et al. Temporal trends in gender-affirming surgery among transgender patients in the United States. JAMA Surg 2018;153(7):609–616; doi: 10.1001/jamasurg.2017.6231 29490365 PMC5875299

[2] Nolan IT, Kuhner CJ, Dy GW. Demographic and temporal trends in transgender identities and gender confirming surgery. Transl Androl Urol 2019;8(3):184–190; doi: 10.21037/tau.2019.04.09 31380225 PMC6626314

[3] Genital Surgery Open Letter to WPATH. Available from: https://wpathopenletter.wordpress.com [Last accessed: July 7, 2022].

[4] Decision Memo for Gender Dysphoria and Gender Reassignment Surgery (CAG-00446N). Centers for Medicare & Medicaid Services. 2016. Available from: https://www.cms.gov/medicare-coverage-database/view/ncacal-decision-memo.aspx?proposed=N&NCAId=282 [Last accessed: July 7, 2022].

[5] White Hughto JM, Reisner SL. A systematic review of the effects of hormone therapy on psychological functioning and quality of life in transgender individuals. Transgend Health 2016;1(1):21–31; doi: 10.1089/trgh.2015.0008 27595141 PMC5010234

[6] Baker KE. The future of transgender coverage. N Eng J Med 2017;376(19):1801–1804; doi: 10.1056/NEJMp1702427 28402247

[7] Horbach SE, Bouman MB, Smit JM, et al. Outcome of vaginoplasty in male-to-female transgenders: A systematic review of surgical techniques. J Sex Med 2015;12(6):1499–1512; doi: 10.1111/jsm.12868 25817066

[8] Andréasson M, Georgas K, Elander A, et al. Patient-reported outcome measures used in gender confirmation surgery: A systematic review. Plast Recon Surg 2018;141(4):1026–1039; doi: 10.1097/prs.0000000000004254 29595738

[9] Dy GW, Nolan IT, Hotaling J, et al. Patient reported outcome measures and quality of life assessment in genital gender confirming surgery. Transl Androl Urol 2019;8(3):228–240; doi: 10.21037/tau.2019.05.04 31380229 PMC6626309

[10] Klaiman T, Pracilio V, Kimberly L, et al. Leveraging effective clinical registries to advance medical care quality and transparency. Popul Health Manag 2014;17(2):127–133; doi: 10.1089/pop.2013.0021 24152057

[11] Jacobs JP, Shahian DM, Prager RL, et al. Introduction to the STS national database series: Outcomes analysis, quality improvement, and patient safety. Ann Thorac Surg 2015;100(6):1992–2000; doi: 10.1016/j.athoracsur.2015.10.060 26525868

[12] Leppke S, Leighton T, Zaun D, et al. Scientific registry of transplant recipients: Collecting, analyzing, and reporting data on transplantation in the United States. Transplant Rev 2013;27(2):50–56; doi: 10.1016/j.trre.2013.01.002 23481320

[13] Rowell KS, Turrentine FE, Hutter MM, et al. Use of national surgical quality improvement program data as a catalyst for quality improvement. J Am Coll Surg 2007;204(6):1293–1300; doi: 10.1016/j.jamcollsurg.2007.03.024 17544087

[14] Frank L, Basch E, Selby JV, et al. The PCORI perspective on patient-centered outcomes research. JAMA 2014;312(15):1513–1514; doi: 10.1001/jama.2014.11100 25167382

[15] Chang HC, Tzou DT, Usawachintachit M, et al. Rationale and design of the registry for stones of the kidney and ureter (ReSKU): A prospective observational registry to study the natural history of urolithiasis patients. J Endourol 2016;30(12):1332–1338; doi: 10.1089/end.2016.0648 27758162 PMC5144847

[16] Spade D. Mutilating gender. In: The Transgender Studies Reader. (Stryker SW, Stephen. eds.) Routledge: New York, NY, USA; 2006.

[17] Stone S. The empire strikes back: A posttranssexual manifesto. Camera Obscura 1992;10:150–176.

[18] Klassen AF, Kaur M, Johnson N, et al. International phase I study protocol to develop a patient-reported outcome measure for adolescents and adults receiving gender-affirming treatments (the GENDER-Q). BMJ Open 2018;8(10):e025435; doi: 10.1136/bmjopen-2018-025435 PMC6196938 30344182

[19] Weinfurt KP, Lin L, Bruner DW, et al. Development and initial validation of the PROMIS(^®^) sexual function and satisfaction measures version 2.0. J Sex Med 2015;12(9):1961–1974; doi: 10.1111/jsm.12966 26346418

[20] Joosten YA, Israel TL, Williams NA, et al. Community engagement studios: A structured approach to obtaining meaningful input from stakeholders to inform research. Acad Med 2015;90(12):1646–1650; doi: 10.1097/acm.0000000000000794 26107879 PMC4654264

[21] Harris PA, Taylor R, Thielke R, et al. Research electronic data capture (REDCap)—A metadata-driven methodology and workflow process for providing translational research informatics support. J Biomed Inform 2009;42(2):377–381; doi: 10.1016/j.jbi.2008.08.010 18929686 PMC2700030

[22] Holly BK, Tracy LT, Bauerband LA. Measuring transgender individuals' comfort with gender identity and appearance: Development and validation of the transgender congruence scale. Psych Women Quart 2012;36(2):179–196; doi: 10.1177/0361684312442161

[23] Levis B, Sun Y, He C, et al. Accuracy of the PHQ-2 alone and in combination with the PHQ-9 for screening to detect major depression: Systematic review and meta-analysis. JAMA 2020;323(22):2290–2300; doi: 10.1001/jama.2020.6504 32515813 PMC7284301

[24] Hays RD, Spritzer KL, Schalet BD, et al. PROMIS(^®^)-29 v2.0 profile physical and mental health summary scores. Qual Life Res 2018;27(7):1885–1891; doi: 10.1007/s11136-018-1842-3 29569016 PMC5999556

[25] Herbenick D, Reece M. Development and validation of the female genital self-image scale. J Sex Med 2010;7(5):1822–1830; doi: 10.1111/j.1743-6109.2010.01728.x 20233278

[26] Cella D, Yount S, Rothrock N, et al. The patient-reported outcomes measurement information system (PROMIS): Progress of an NIH roadmap cooperative group during its first two years. Med Care 2007;45(5 Suppl. 1):S3–S11; doi: 10.1097/01.mlr.0000258615.42478.55 PMC2829758 17443116

[27] Barry MJ, Fowler FJ Jr., O'Leary MP, et al. The American Urological Association symptom index for benign prostatic hyperplasia. J Urol 2017;197(2s):S189–S197; doi: 10.1016/j.juro.2016.10.071 28012747

[28] Uebersax JS, Wyman JF, Shumaker SA, et al. Short forms to assess life quality and symptom distress for urinary incontinence in women: The Incontinence Impact Questionnaire and the Urogenital Distress Inventory. Continence Program for Women Research Group. Neurourol Urod 1995;14(2):131–139; doi: 10.1002/nau.1930140206 7780440

[29] Barone M, Cogliandro A, Di Stefano N, et al. A systematic review of patient-reported outcome measures following transsexual surgery. Aesthetic Plast Surg 2017;41(3):700–713; doi: 10.1007/s00266-017-0812-4 28204933

[30] Clennon EK, Martin LH, Fadich SK, et al. Community engagement and patient-centered implementation of patient-reported outcome measures (PROMs) in gender affirming surgery: A systematic review. Curr Sex Health Rep 2022;14(1):17–29; doi: 10.1007/s11930-021-00323-6

[31] Chang EM, Gillespie EF, Shaverdian N. Truthfulness in patient-reported outcomes: Factors affecting patients' responses and impact on data quality. Patient Relat Outcome Meas 2019;10:171–186; doi: 10.2147/prom.S178344 31354371 PMC6573779

[32] Franklin P, Chenok K, Lavalee D, et al. Framework to guide the collection and use of patient-reported outcome measures in the learning healthcare system. EGEMS (Washingdon, DC) 2017;5(1):17–17; doi: 10.5334/egems.227 PMC5983040 29881737

[33] Dy GW, Blasdel G, Downing JM. Centering transgender and nonbinary voices in genital gender-affirming surgery research prioritization. JAMA Surg 2022;157(7):628–629; doi: 10.1001/jamasurg.2022.1049 35544129 PMC9395165

[34] Huber S, Ferrando C, Safer JD, et al. Development and validation of urological and appearance domains of the post-affirming surgery form and function individual reporting measure (AFFIRM) for transwomen following genital surgery. J Urol 2021:101097ju0000000000002141; doi: 10.1097/ju.0000000000002141 34288738

